# Surgical Treatment of Goitre

**Published:** 1880-03

**Authors:** 


Surgical Treatment of Goitre. — Dr. Wolfler, in speaking of the treatment of goitre with subcutaneous injections of iodine, says (Langenbeck's Bd. 24, Heft. 1), that favorable results have been obtained both in cases of simple hyperplasia, and a colloid degeneration. He illustrates his statements by a few cases from Billroth's clinic, and an experiment on a dog made by himself. The lobes of the thyroid gland of this dog had respectively attained the size of a goose's egg, and the author made ten injections of iodine into one of the lobes. The dog was killed at the end of a month, when the portion of the goitre into which the injections had been made was found to have dwindled down to the size of a man's thumb; it consisted of connective tissues which no longer contained any colloid liquid. The peripheric part of the injected goitre presented the same appearance as the lobe which had remained untouched; it consisted of large meshes of connective tissue, which contained colloid fluid. There were no traces of inflammation or hemorrhage following the injection of iodine. Several strumous cysts were treated in a different manner; one cyst with thin walls was absobed after injections of iodine; two other cysts resisted this treatment. In two cases Billroth drained strumous cysts with antiseptic precautions. In one of these cases, the cure was speedily effected; in the other, the cyst was wholly absorbed, as there were calcareous deposits in its walls. The sac was then opened and the contents removed, after which the patient, a woman aged seventy-two, recovered. The author thinks that tapping the cyst and putting in a drainage tube ought to be done in cases where a cyst does not collapse immediately after being tapped, or in old people where the injection of iodine might be succeeded by a strong reaction, but where the extirpation of the goitre might prove fatal. In the course of the last year, Billroth has extirpated goitres in seven cases under antiseptic precautions, the results having each time been very favorable. In one of these cases the patient was suffering from malignant cystic papilloma; in another case the struma was of carcinomatous nature. All the wounds healed by first intention.—*London Med. Record.*

